# Gamma irradiation and ozone application as preservation methods for longer-term storage of bee pollen

**DOI:** 10.1007/s11356-024-32801-4

**Published:** 2024-03-11

**Authors:** Yahya Al Naggar, Ibrahim M. Taha, El-Kazafy A. Taha, Ayman Zaghlool, Ali Nasr, Ashraf Nagib, Sam M. Elhamamsy, Gomaa Abolaban, Alaa Fahmy, Eslam Hegazy, Khaled H. Metwaly, Abdullah A. Zahra

**Affiliations:** 1 Zoology Department, Faculty of Science, Tanta University, Tanta, 31527 Egypt; 2 Department of Food Science and Technology, Faculty of Agriculture, Al-Azhar University, Nasr City, Cairo 11884 Egypt; 3 Department of Economic Entomology, Faculty of Agriculture, Kafr Elsheikh University, Kafr Elsheikh, 33516 Egypt; 4 Department of Biochemistry, Faculty of Agriculture, Al-Azhar University, Nasr City, Cairo 11884 Egypt; 5 Department of Plant Protection, Faculty of Agriculture, Al-Azhar University, Nasr City, Cairo 11884 Egypt; 6 Chemistry Department, Faculty of Science, Al-Azhar University, Cairo, 11884 Egypt; 7 Petrochemicals Department, Faculty of Engineering, Pharos University in Alexandria, Alexandria, Egypt; 8 Department of Food Irradiation, National Centre for Radiation Research and Technology (NCRRT), Egyptian Atomic Energy Authority (EAEA), Cairo, 11787 Egypt; 9 Center of Plasma Technology, Al-Azhar University, Cairo, 11884 Egypt; 10 Center of Bee Research and its Products, King Khalid University, P.O. Box 9004, Abha, 61413 Saudi Arabia

**Keywords:** Ozone, Gamma irradiation, Preservation methods, Bee pollen, Microbial load

## Abstract

Bee pollen is a healthy product with a good nutritional profile and therapeutic properties. Its high moisture content, however, promotes the growth of bacteria, molds, and yeast during storage commonly result in product degradation. Therefore, the aim of this study is to assess the effectiveness of gamma irradiation (GI) and ozone (OZ) as bee pollen preservation methods for longer storage time, as well as whether they are influenced by pollen species. To do that, GI at a dosage of 2.5, 5.0, and 7.5 kGy was applied at a rate of 0.68 kGy/h and OZ application at a concentration of 0.01, 0.02, and 0.03 g/m^3^ was applied for one time for 6 h, to Egyptian clover and maize bee pollen, then stored at ambient temperature for 6 months. We then determined the total phenolic content (TPC) and antioxidant activity of treated and non-treated pollen samples at 0, 3, and 6 months of storage. Total bacteria, mold, and yeast count were also evaluated at 0, 2, 4, and 6 months. Statistical analyses revealed that, TPC, antioxidant, and microbial load of both clover and maize pollen samples were significantly (*p* < 0.05) affected by both treatment and storage time and their interaction. Both methods were extremely effective at preserving the antioxidant properties of pollen samples after 6 months of storage at room temperature. Furthermore, the highest concentrations of both GI and OZ applications completely protected pollen samples from mold and yeast while decreasing bacterial contamination. GI at the highest dose (7.5 KGy) was found to be more effective than other GI doses and OZ application in preserving biologically active compounds and lowering the microbial count of pollen samples for 6 months. As a result, we advise beekeepers to use GI at this dose for longer-term storage.

## Introduction

Consumers use bee pollen as a supplement to attain specific health effects. Bee pollen medicinal capabilities have been utilized for thousands of years (Xi et al. 2018; Nainu et al. 2021; Algethami et al. 2022; El-Seedi et al. 2022). It is full of nutrients and phytochemicals (Campos et al. 2008). Unfortunately, bee pollen is also an optimum medium for the growth and multiplication of bacteria, molds, and yeasts. Microbiological load is the most important quality criterion for bee pollen. Contaminants can reach the pollen from plants, water, soil, and air (Taha et al. 2017). Bee’s activities, human actions (handling, collecting, drying, and packaging) and environmental conditions may act as a microbial contamination source (Beev et al. 2018). Pollen is contaminated by bacteria through many sources including honey bee digestive systems, dust, nectar, and ground, whereas post-harvest bacterial sources include people, tools, containers, pests, and water (López et al. 2020). Bee pollen has been reported to contain both bacteria and fungi, demonstrating that it provides an ideal habitat for microbial growth. Microorganisms in bee pollen can cause safety issues or a reduction in shelf-life. Therefore, a common practice before commercialization is the application of preservation processes to increase bee pollen shelf-life and safety (Mekki 2018).

Gamma irradiation (GI) has been approved as a safe method for storing products (Nasr et al. 2022). Irradiation can be considered a successful method for reducing the prevalence of food-borne diseases and for treating several possible issues in food (Sajjabut et al. 2019). More than 60 products are being irradiated worldwide thanks to this technology, which has received approval in more than 50 countries, including Egypt (Maherani et al. 2016). Many agricultural issues have been successfully resolved by irradiation resulting in a decrease in post-harvest losses and the improvement of salt stress tolerance (Villavicencio et al. 2018)

Ozone (OZ) is a potent oxidant and antibacterial agent that can be used to sterilize germs in the food business (Reddy et al. 2021; Kaavya et al. 2021). Ozone is an extremely reactive gaseous molecule. It can be created using UV radiation or electric discharge (Sivaranjani et al. 2021). It has considered to be a very unstable with a half-life time of roughly 22 min at 20 °C since it has produced immediately from freely available air (Fahmy et al. 2013; Pandiselvam et al. 2020).

Bee pollen is collected in large quantities throughout the seasons, and due to its nutritional value, it has been recommended as a nutritious food for humans (Taha 2015; Taha et al. 2019; Al-Kahtani et al. 2020, 2021; Al-Kahtani and Taha 2021); as a result, it should be preserved in suitable conditions to avoid deterioration until it is employed for various purposes, whether nutritional or medicinal. Unfortunately, it can be contaminated with aerobic bacteria, molds, and yeasts from natural habitats, human activities, and environmental factors leading to decrease their storage period. Previous studies investigated several methods for conserving bee pollen and other hive products. For example, ethanol and silica gel have been investigated as preservation strategies for honey bee-collected pollen metabarcoding (Quaresma et al. 2021). Drying, pasteurization, and high-pressure pasteurization were also investigated as methods for preservation (Anjos et al. 2023). In addition, OZ and GI exposure have been employed as bee pollen preservation techniques, though only for short periods (Álvarez Hidalgo et al. 2020; Cabello et al. 2021). As a result, their efficacy for longer-term preservation should be validated, as well as whether they are influenced by pollen species or not.

Therefore, this study aims to gain insight into the efficacy of GI and OZ applications for longer-term bee pollen preservation, as well as changes in bioactive components and microbial contamination of bee pollen during storage.

## Materials and methods

### Chemicals

2,2-Diphenyl-1-picrylhydrazyl (DPPH), gallic acid, Folin-Ciocalteu’s phenol reagent, and aluminum chloride were purchased from Sigma Company, USA and Al-Nasr Company, Cairo, Egypt. All reagents and chemicals were analytical grade.

### Bee pollen

Clover (*Trifolium alexandrinum* L.) and maize (*Zea mays* L.) have been reported as major pollen sources in Egypt (Taha et al. 2019), so they were selected for this study. Clover and maize bee pollens were collected from the Basyuon district (30°56′31.1″N, 30°48′41.5″E) Gharbia Governorate, Egypt during year 2022. To do that, five honey bee colonies from hybrid Carniolan honey bees (*Apis mellifera carnica* Pollmann) were used to collect bee pollen. Standard traps were fixed on the hive entrance to trap pollen loads of clover during May and maize during August. The traps were harvested daily, and the collected pollen was cleaned and separated based on the color. The collected pollen was kept at − 20 °C in polyethylene bags until delivery to the laboratory for further tests. For the preservation tests, 1 kg of pollen loads of each botanical origin was used.

### Preservation methods

The bee pollen samples were packed into polyethylene zip bags (30 g/bag). Packaged bee pollen was exposed to different GI doses (2.5, 5.0, and 7.5 kGy) at the rate of 0.68 kGy/h. Irradiation treatments were performed at the National Center for Radiation and Technology (NCRRT), Atomic Energy Authority, Egypt, using 60 Co γ-ray (Indian irradiation unit). While OZ was pumped into the container from ozone generator (Tianjin, China), and the concentration was kept at 0.01, 0.02, and 0.03 g/m^3^. Then, pollen jars were exposed for 6 h, and an OZ analyzer was utilized to determine the OZ content in jars (H1-AFX Instrumentation, USA).

**Fig. 1:**
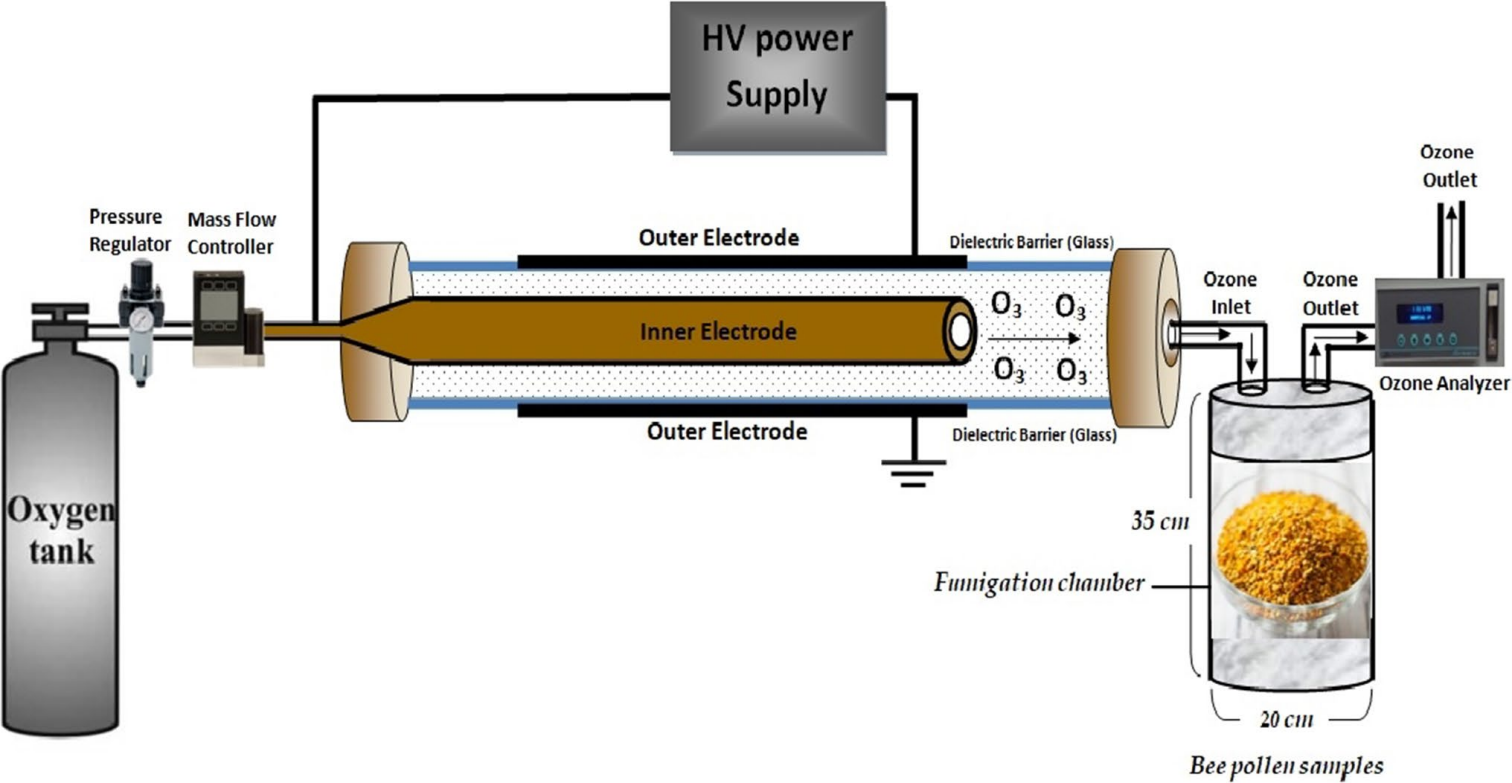
Schematics diagram of ozone generation for bee pollen treatment

Oxygen was used as an input in the gas generation process for the OZ application, which went through a dielectric barrier discharge reactor. This type of discharge is produced by applying a discharge voltage between two coaxial electrodes, having a glass dielectric between them and a free space where the oxygen flows through. In this free space, a filament discharge is produced, where electrons are generated with enough energy to breakdown the oxygen molecules forming OZ. Figure 1 reveals the diagram of OZ generation. The flow rates of oxygen are fixed to 0.1 L min^−1^ to generate OZ molecules. The voltage of the AC test was set to 220 V at 50 Hz, and the voltages were controlled by a transformer control box (Variac Variable AC Power Transformer Regulator). Ozone (O_3_) is formed when O_2_ molecules are dissociated by electron impact. Table 1 shows the applied voltage and corresponding power values at different OZ concentrations.

**Table 1:** Applied voltage and corresponding power values at different ozone concentrations

Ozone concentration (g/m^3^)	Applied voltage (kV)	Electric power P_el_ (Watts)
0.01	2.6	0.74
0.02	3.5	1.04
0.03	4.3	1.7

### Phytochemical contents

The extraction was conducted as describe by (Keskin and Özkök 2020). A 3 g of bee pollen was mixed with 20 mL of ethanol using mechanical shaker for 12 h, then centrifuged at 3000 rpm for 15 min (HERMLE, Z326K), then filtered and kept at 4 °C. The extracts were used to assess total phenolic contents and antioxidant activity in pollen samples immediately after treatment with various concentrations of GI or OZ application, as well as 3 and 6 months later. In each treatment and time point, three samples were used.

### Total phenolics content (TPC)

The Folin-Ciocalteu method has been followed according to (Amarasinghe et al. 2021). A 2.5 mL (10 %) of Folin-Ciocalteu reagent mixed with 0.5 mL of bee pollen extract. The mixtures were kept for 8 min in the dark; then, 2 mL of Na_2_CO_3_ (7.5%) was added and mixed well. The solution was incubated in the dark for 60 min. Absorbances were measured by spectrophotometry (Cary 50 Bio Varian, Australia) at 765 nm. The result was calculated as mg Gallic 100 g^−1^ of sample.

### Antioxidant activities

The antioxidant activities of bee pollen samples were evaluated as reported by (Sakooei-Vayghan et al. 2020). A 2 mL DPPH solution (0.16 mM) was vortexed with 2 mL of bee pollen extract, then incubated in darkness for 30 min. Absorbance reads spectrophotometry at 517 nm were recorded. The % of inhibition was calculated from the equation:

$$\% of\ DPPH\ Inhibition=\frac{\Big(\left( control\ absorbance- sample\ absorbance\right)}{Control\ absorbance\Big)\ }\ X\ 100$$

### Microbial evaluation

The method of Estevinho et al. (2012) was used for microbiological examination. Plate agar (PCA) was used to count total bacteria, which was cultured for 48 hours at 35 °C. To count yeasts and molds, PDA media were employed, and after 3 to 5 days at 25 °C. The microbial counts were recorded as Log colony-forming unit per gram (CFU g^−1^) after 0, 2, 4, and 6 months of storage at room temperature. In each treatment and time point, three samples were used.

### Statistical analysis

GraphPad Prism 8.00 for Windows was used for data analysis and visualization (www.graphpad.com). Normality of data was assessed by use of the Kolmogorov-Smirnov test, and homogeneity of variance was assessed by use of Levene’s test. To compare the change in TPC, antioxidant activities, and microbial counts (bacteria, mold, and yeast) between treated and non-treated pollen samples at different time points, we used a two-way RM analysis of variance (ANOVA). To test for significant interactive effects of exposure to either GI or OZ treatments, and time of assessment of investigated endpoints, we inspected the treatment × time interaction terms in all tests followed by a pairwise Tukey post hoc test with Bonferroni correction for multiple comparisons. A significance level of 0.05 was used to define a test’s significance.

## Results

### Effects on total phenolics content and antioxidant activities

**Fig. 2:**
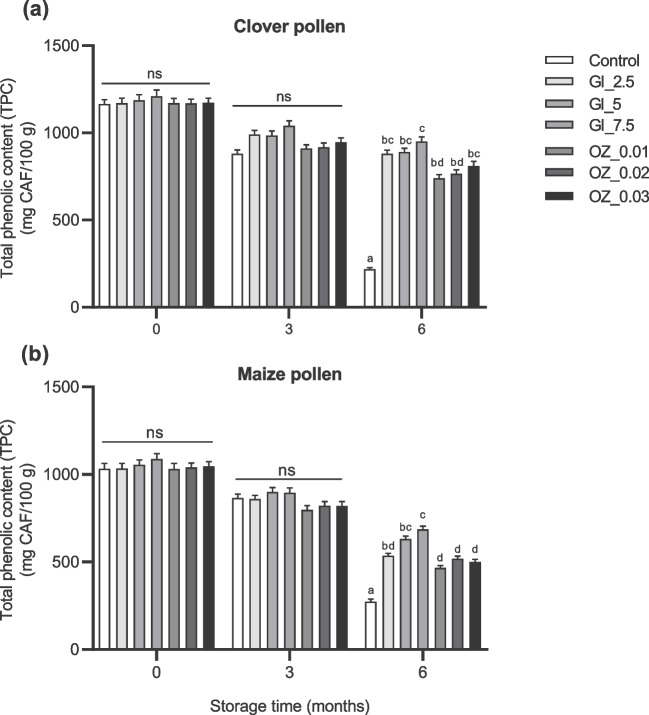
Total phenolic contents (TPC) in **a** clover and **b** maize pollen samples after 0, 3, and 6 months of storage at room temperature. Pollen samples were gamma irradiated (GI) at various doses (2.5, 5, and 7.5 kGy) or treated with ozone (OZ) at various concentrations (0.01, 0.02, and 0.03 g/m^3^) for one time before being stored. Column bars (mean ± SEM, *n* = 3 samples) with different lowercase letters denote significant differences among treatments at each time point (two way-ANOVA, *p* < 0.05, a posteriori Tukey test with Bonferroni correction for multiple comparisons). ns (not significant)

When we compared the effects of GI and OZ application immediately after application and 3 and 6 months later, we found significant treatment x time interaction terms, indicating that both TPC and antioxidant activity of pollen samples changed over time because of treatment (Table 2). There were no significant effects on TPC after 0 and 3 months of storage between control and treated pollen samples. After 6 months, however, treated clover and maize pollen samples had 70–80% and 50–60% of their initial TPC, respectively, compared to control clover, which had 18% and maize pollen, which had 26% of their initial TPC (Fig. 2). Clover and maize pollen samples treated with the highest dose of GI (7.50 kGy) demonstrated significantly higher TPC than other treated pollen samples (Fig. 2).

**Table 2:** Results from statistical two-way RM ANOVA analyses testing effects of three different doses of gamma irradiation (GI) and three different ozone (OZ) concentrations on total phenolic content (TPC), antioxidant activity (% DPPH), total anaerobic bacterial count (TBC), and mold and yeast count at different time points

Source	Two-way RM ANOVA		
	df	*p* value	
**TFC**		Clover	Maize
Treatment	6	< 0.001	0.002
Time	2	< 0.001	< 0.001
Treatment × time	12	< 0.001	< 0.001
**% DPPH**			
Treatment	6	0.001	0.001
Time	2	< 0.001	< 0.001
Treatment × time	12	< 0.001	< 0.001
**TBC**			
Treatment	6	< 0.001	< 0.001
Time	3	< 0.001	< 0.001
Treatment × time	6	< 0.001	< 0.001
**Mold and yeast**			
Treatment	6	< 0.001	< 0.001
Time	3	< 0.001	< 0.001
Treatment × time	6	< 0.001	< 0.001

**Fig. 3:**
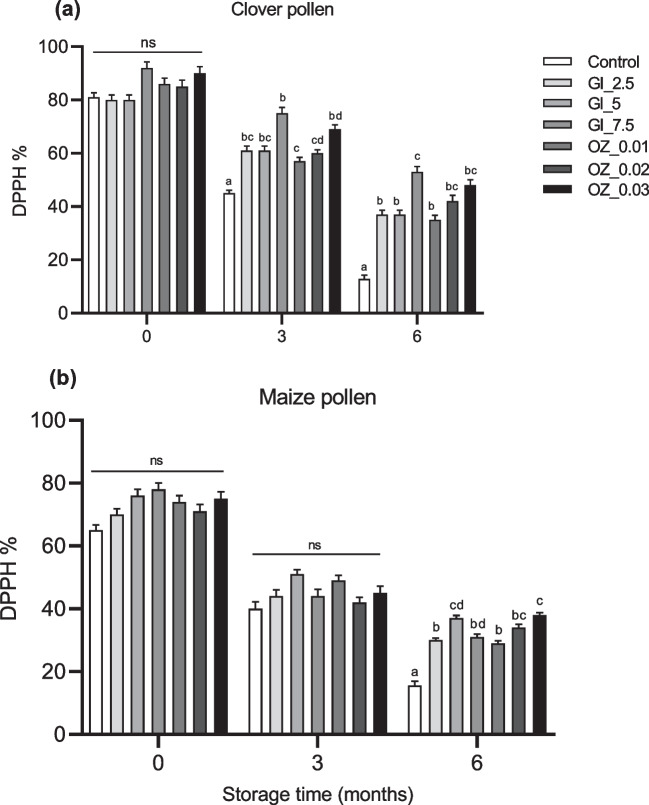
DPPH % in **a** clover and **b** maize pollen samples after 0, 3, and 6 months of storage at room temperature. Pollen samples were gamma irradiated (GI) at various doses (2.5, 5, and 7.5 kGy) or treated with ozone (OZ) at various concentrations (0.01, 0.02, and 0.03 g/m^3^) for one time before being stored. Column bars (mean ± SEM, *n* = 3 samples) with different lowercase letters denote significant differences among treatments at each time point (two way-ANOVA, *p* < 0.05, a posteriori Tukey test with Bonferroni correction for multiple comparisons). ns (not significant)

There were no significant changes (*p* > 0.05) in antioxidant activity measured as % of DPPH immediately after treatment with either GI or OZ application between control and treated pollen samples. However, after 3 months of room temperature storage, there was a significant decrease (*p* < 0.05) in the percentage DPPH of non-treated clover pollen samples (control) compared to treated samples (Fig. 3a), but no significant changes (*p* > 0.05) in antioxidant activity of treated maize pollen samples compared to control samples (Fig. 3b).

After 6 months of storage, treated clover and maize pollen samples retained 45–55% of their antioxidant activity when compared to control clover and maize pollen samples, which had 15–23% of their initial antioxidant activity (Fig. 3). Clover pollen samples treated with the highest dose of GI (7.50 kGy) demonstrated significantly (*p* < 0.05) higher antioxidant than other irradiated pollen samples (Fig. 2a).

### Effects on microbial counts

**Fig. 4:**
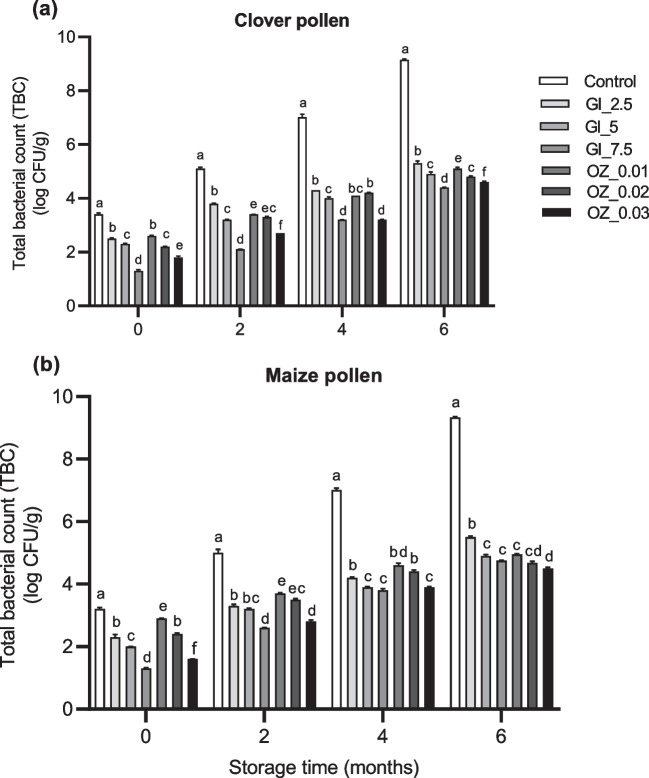
Total bacterial count (TBC) in **a** clover and **b** maize pollen samples after zero and two months of storage at room temperature. Pollen samples were gamma irradiated (GI) at various doses (2.5, 5, and 7.5 kGy) or treated with ozone (OZ) at various concentrations (0.01, 0.02, and 0.03 g/m^3^) for one time before being stored. Column bars (mean ± SEM, *n* = 3 samples) with different lowercase letters denote significant differences among treatments at each time point (two way-ANOVA, *p* < 0.05, a posteriori Tukey test with Bonferroni correction for multiple comparisons)

**Fig. 5:**
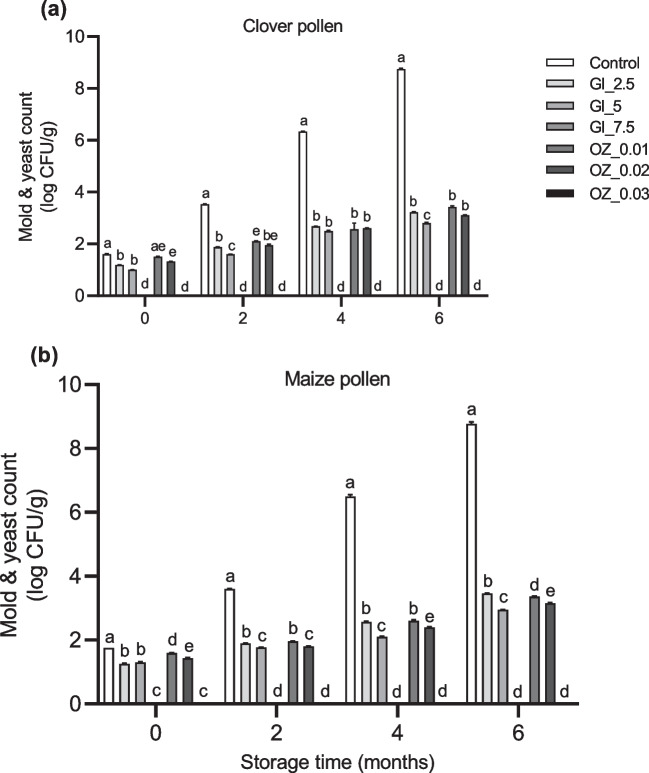
Mold and yeast count in **a** clover and **b** maize pollen samples after 0 and 2 months of storage at room temperature. Pollen samples were gamma irradiated (GI) at various doses (2.5, 5, and 7.5 kGy) or treated with ozone (OZ) at various concentrations (0.01, 0.02, and 0.03 g/m^3^) for one time before being stored. Column bars (mean ± SEM, *n* = 3 samples) with different lowercase letters denote significant differences among treatments at each time point (two way-ANOVA, *p* < 0.05, a posteriori Tukey test with Bonferroni correction for multiple comparisons)

We compared the effects of different concentrations of GI and OZ application after 0, 2, 4, and 6 months of storage on microbial counts, we found significant (*p* < 0.05) treatment × time interaction terms, indicating that both TBC and mold and yeast in pollen samples changed over time due to treatment (Table 2). Interestingly, after immediately exposing both clover and maize pollen samples to GI or OZ treatments and then storing them at room temperature for 2, 4, and 6 months, a dose-dependent decrease in TBC, mold, and yeast was observed (Figs. 4 and 5). Additionally, we observed no mold or yeast counts in both pollen species that had been exposed to the highest dose or concentration of GI or OZ application at 0, 2, 4, and 6 months of storage at room temperature (Fig. 5), indicating the great efficiency of both preservation methods at these high levels against mold and yeast contamination.

## Discussion

Pollen is the most important source of amino acids, carbohydrates, lipids, vitamins, enzymes, and minerals (Algethami et al. 2022); however, it additionally acts as a vehicle for the spread of viruses, bacteria, and fungi (Disayathanoowat et al. 2020). Fresh pollen is difficult to store due to its high-water content, so it must be dried and sterilized. We tested the efficacy of two sterilization methods on clover and maize pollen using different concentrations of GI and OZ treatments. Both methods were extremely effective at preserving the antioxidant properties of pollen samples for 6 months of storage at room temperature. Furthermore, the highest concentrations of both GI and OZ application protected pollen samples from mold and yeast while decreasing bacterial contamination.

In recent years, numerous studies on the antioxidant potential of foods and food supplements, including bee pollen, have been carried out. Numerous studies have shown that bee products, including pollen, have high antioxidant activity, and contain significant amounts of natural antioxidants (Mayda et al. 2020; Tutun et al. 2021; Durazzo et al. 2021; Gercek et al. 2021; Sawicki et al. 2022; Straumite et al. 2022). Bee pollen’s antioxidant capacity varies according to the plant from which it is derived, as well as the geographical and climatic characteristics of the region in which it is provided (Araújo et al. 2017; Kostić et al. 2017). In the current study, GI- and OZ-exposed clover and maize pollen samples retained ~ 50–80% of their TPC and antioxidant activity, when compared to non-treated clover and maize pollen samples, which had 15–26% of their initial TPC and antioxidant after 6 months of room temperature storage. Previous research found that GI treatment stimulated phenylalanine ammonia lyase, maintained the highest level of total phenolic content of fresh *Lentinula edodes* during cold storage, and postponed the occurrence of reduced ascorbic acid, which contributed to strengthening the antioxidant capacity (Fan et al. 2003; Cheng et al. 2023; Shi et al. 2022). Furthermore, phenolic compounds are increased as a result of changes in enzyme concentration and activity caused by ozone (Sachadyn-Król and Agriopoulou 2020). Our findings are therefore consistent with previous studies that reported similar results on fresh fruit and vegetables using GI (Beaulieu et al. 2002; Breitfellner et al. 2002; Fan et al. 2003; Song et al. 2006; Shahi et al. 2021) and OZ exposure (reviwed in, Sachadyn-Król and Agriopoulou 2020).

When pollen is collected without being dried or processed, the growth of microorganisms can compromise pollen quality and storage time, resulting in negative side effects such as fermentation or mycotoxin production (Nogueira et al. 2012; Fatrcová-Šramková et al. 2016; Mauriello et al. 2017). The microbiological load of bee pollen was significantly influenced by GI and OZ treatments in the current study. When compared to non-treated pollen samples, which had a more than twofold increase in bacterial load over the 6-month storage period at room temperature, the highest concentrations of both GI and OZ application protected pollen samples from mold and yeast while decreasing bacterial contamination. The latter could be attributed to the loss of TPC and antioxidant activity in non-treated pollen, because antioxidant activity and phytochemical content significantly correlate with antimicrobial activity (Groppo et al. 2011; Azeez et al. 2012; Todorovic et al. 2017; Suleiman and Ateeg 2020). For example, phenolic, flavonoid, carotene, and free radical scavenging capacity contents of decaying spices have been found to be significantly lower than those of fresh spices (Azeez et al. 2012). The time of exposure to either OZ or GI is also important. Previous research discovered that ozonation at a density of 200 mg/h for a short time (30–60 min) preserved a low microbial load in bee pollen during 6 weeks of storage under refrigeration (Cabello et al. 2021). As a result, ozonation for 6 h, as investigated in the current study, should be followed because it protected pollen samples from mold and yeast while decreasing bacterial contamination after 6-month storage at room temperature.

In the current study, we noticed that GI had a better sterilization effect on both clover and maize pollen samples, especially at the highest dose, a finding that is consistent with a recent study that found that pollen treated with GI and ethylene oxide application had higher pathogen suppression than pollen treated with OZ application (Strange et al. 2023). GI sterilization has also demonstrated broad applicability for a variety of pharmaceutical products (Hasanain et al. 2014). When GI bee pollens and those treated with OZ application at the highest doses are compared to non-treated pollen samples after 6 months of room temperature storage, it is interesting to note that they do not exceed the limits for total aerobic bacteria (100,000 CFU/g) recommended by the European Union standard for microbiological quality (Campos et al. 2008). This shows the significance of GI and OZ application in suppressing microbial growth and prolonging the shelf-life of pollen.

## Conclusions

Bee pollens are often characterized by their phenolic and antioxidant content. GI and OZ treatments enhanced the microbial safety of bee pollens and reduced the microbial load which is critical for food security and human health. It is also worth noting that, regardless of pollen species, both preservation methods produced comparable results. However, GI at the highest dose (7.5 KGy) was found to be more effective than other GI doses and OZ application in preserving biologically active compounds and lowering the microbial count of pollen samples for 6 months. As a result, we recommend that beekeepers use GI at this dose and, if possible, store it under vacuum for longer periods of time. Future research should identify and quantify the bioactive components of GI- or OZ-exposed pollen samples during long-term storage to understand more about their involvement in maintaining TPC while decreasing pollen microbial load.

## Data Availability

The datasets generated during and/or analyzed during the current study are available from the corresponding author on reasonable request.
